# Implementation outcomes of peer education programme comparing state-led and NGO-facilitated models in two Indian states: qualitative findings

**DOI:** 10.3389/fpubh.2024.1434959

**Published:** 2024-11-01

**Authors:** Monika Arora, Shalini Bassi, Deepika Bahl, Nishibha Thapliyal, Deepak Kumar, Harish K. Pemde, Zoya Ali Rizvi

**Affiliations:** ^1^Health Promotion Division, Public Health Foundation of India (PHFI), New Delhi, India; ^2^Ministry of Health and Family Welfare, Nirman Bhavan, Government of India, New Delhi, India; ^3^Department of Pediatrics, Lady Hardinge Medical College, Kalawati Saran Children's Hospital, New Delhi, India

**Keywords:** peer education, adolescent, National Adolescent Health Strategy, RKSK, peer educators

## Abstract

**Introduction:**

Each Indian state can select one of the two implementation models under India’s National Adolescent Health Strategy, i.e., Rashtriya Kishor Swasthya Karyakram, either direct implementation through the existing State Health Department and systems, or the Non-Governmental Organisation (NGOs) implementation model, which involves partnering with one or more field-level NGOs to provide the services and personnel.

**Methods:**

To compare and comprehend the implementation strategies of the Peer Education programme under the Direct and NGO implementation models within India’s National Adolescent Health Strategy, and to document factors facilitating and hindering the adoption and implementation of the programme across two Indian states, using a qualitative approach.

**Results:**

Variations and similarities were seen across the two models. Employing a multi-level selection process, Madhya Pradesh selected two peer educators (PEs), while Maharashtra had four. Criteria included adolescents aged 15 and above in Madhya Pradesh and younger (10–14 years) and older (15–19 years) in Maharashtra. Madhya Pradesh selected Shadow Peers (10–14 years) to address attrition. Training in Madhya Pradesh spanned over 6 days, structured, led by NGO Mentors, utilising standardised, interactive resources with participatory methods. Maharashtra’s training, facilitated by Auxiliary Nurse Midwife or Medical Officer, followed traditional approaches and relied on the trainer’s expertise. PE session frequency and duration varied from monthly to quarterly. PEs were comfortable in handling issues like nutrition and non-communicable diseases but faced hesitancy in handling sexual and reproductive health issues. Regular Adolescent Friendly Clubs supported peer educators (PEs). In Madhya Pradesh, Adolescent Health and Wellness Days were suspended due to the pandemic, which led to decreased awareness of adolescent health services. Maharashtra resumed Adolescent Health and Wellness Days albeit on a limited scale.

**Conclusion:**

The study identified various similarities and deviations from operational guidelines for the implementation of the peer education programme, offering valuable guidance for policymakers, practitioners, and stakeholders involved in RKSK’s planning and implementation. It presents actionable strategies to strengthen peer education interventions within national adolescent health programmes, regionally and globally.

## Introduction

Recognising the importance of protecting and investing in adolescents’ health and well-being, the Ministry of Health and Family Welfare, Government of India, launched the National Adolescent Health Strategy (Rashtriya Kishor Swasthya Karyakram or RKSK, 2014), to improve the prospects of adolescent’s crucial physical, biological, and psychological outcomes (1). RKSK prioritises various aspects of adolescent health including Sexual and Reproductive Health, non-communicable diseases, nutrition, mental health, substance misuse and injuries and violence. The programme adopts a comprehensive approach, integrating community and school-based health promotion and prevention efforts while enhancing preventive, diagnostic, and curative services across health facilities (1). A unique and central component of RKSK is the community-based Peer Education programme. The Peer Education programme ensures that adolescents (10–19 years) benefit from regular and sustained peer education to improve the life skills, knowledge and aptitude of adolescents (1). Although RKSK has been in operation since 2014, however on-ground implementation of the peer education programme effectively started after 2017–18.

According to the implementation guidelines of RKSK (1), each state can choose from the two models of peer education programme implementation; i.e. Direct implementation through the existing State Health Department and systems or secondly the Non-Governmental Organisation (NGO)-implementation model by utilising the services and personnel of one or more field-level NGOs. In the direct implementation model, the Accredited Social Health Activist (ASHA), act as the village-level peer educator coordinator. ASHA is a trained female community health activist, selected from the village itself and serves as a crucial interface between the community and the public health system (2). ASHA plays a pivotal role in overseeing the implementation of the peer education programme-related activities at the village level. The Auxiliary Nurse Midwife (ANM) or Male Health Worker facilitate the monthly Adolescent Friendly Club (AFC) sessions, while oversight is provided by the Medical Officer in charge at the Primary Health Centre (PHC). Whereas in the NGO implementation model, well-established field-level NGOs provide supportive supervision for implementing peer education programme activities.

The effectiveness of Peer Education interventions shows variability in global literature (3), with limited evidence from Low- and Middle-Income Countries. In light of this gap, an Implementation Research, i-Saathiya (where “Saathiya” signifies “peer” in English and ‘i’ stands for implementation science) was conducted to compare and comprehend the implementation strategies of the Peer Education programme under the Direct and Non-Governmental Organisation implementation models and document the factors that facilitate and hinder the adoption and implementation of the Peer Education programme under India’s National Adolescent Health Strategy in two Indian States (Madhya Pradesh and Maharashtra). Within the RKSK, the array of community-based approaches encompasses the Weekly Iron Folic Acid Supplementation Programme, Deworming during National Deworming Day, Peer Education programme, provision of sanitary napkins under the Menstrual Hygiene Scheme, AFC and Adolescent Health and Wellness Days (AHWDs). The scope of this paper is confined to Peer Education programme, AFCs and AHWDs. We anticipate that the findings of this study will offer valuable insights not only for India in determining the potential for scaling up this approach to other Indian states but also for countries in the Southeast Asia Region (SEAR) and globally where the Peer Education approach is employed to promote adolescent health and well-being due to the comprehensiveness of India’s peer education programme with its priority themes. These findings will further contribute to the science on the facilitators and barriers of peer education programme implementation in LMICs, as well as the global literature on the effectiveness of Peer Education interventions.

## Methods

### Study setting and participants

The study was cross-sectional and employed a mixed-methods approach, conducted in two Indian states: Madhya Pradesh (NGO implementation model) and Maharashtra (Direct Implementation model). These states differ in implementation models for training peer educators (PEs) and providing supportive supervision for the peer-led sessions. A detailed description of the selection process of the two study states and two districts per state has been described in a prior publication of the study (4). Stakeholders involved in the implementation of the programme at the state, district, block and village levels were recruited for the qualitative study. A total of 234 participants from Madhya Pradesh and Maharashtra were included in the study through In-depth Interviews (IDIs) and Focus Group Discussions (FGDs). The participants were divided nearly evenly between the two states, with 113 participants from Madhya Pradesh and 121 from Maharashtra. The gender distribution was also relatively balanced, with 107 males and 127 females participating in the study. Refer to Table 1 for the study participants’ demographics.

**Table 1:** Demographic characteristics of the study participants.

Participant	State		Gender	
	Madhya Pradesh	Maharashtra	Male	Female
In-depth interviews (IDIs) and Focus Group Discussions (FGDs)				
ASHA	4	4	0	8
ASHA Facilitator	4	4	0	8
ANM	4	4	0	8
CHO	4	4	3	5
NGO Trainer Mentor	4	0	2	2
NGO representative	2	0	2	0
Teacher	4	4	6	2
Peer Educators	12	12	12	12
Adolescents	54	66	64	56
Parent of PE	6	6	4	8
Parent of Adolescent	6	6	4	8
Medical Officer	2	2	3	1
Counsellor	4	4	2	6
Training Faculty	0	2	1	1
State RKSK Coordinator	1	1	2	0
District Coordinator	2	2	2	2
Sub Total	113	121	107	127
	234		234	

### Data collection and analysis

For methodological and data triangulation (5), our study employed a combination of qualitative methods (IDIs, FGDs, and semi-structured observations), alongside surveys (quantitative) and routine programme data. This paper primarily examines the qualitative data and routine programme data. Guides for IDIs, FGDs, and checklists for structured observations, were developed based on the RKSK operational framework (6), implementation guidelines (1) and participants’ roles and responsibilities as allocated under the Peer Education programme (Supplementary Table 1). A data-driven open coding framework was developed for thematic analysis, wherein we systematically arranged, categorised and analysed the data using both inductive and deductive approaches (7). Data from the FGD, IDI and observations were coded deductively and organised thematically according to predefined themes from the qualitative guides. Additionally, in line with inductive principles, new themes were identified based on the information gathered during the interviews, group discussions and observations. An iterative process was used to review the data, identify emerging themes, and refine and develop the final themes and sub-themes throughout the stages of data collection and analysis. The routine RKSK data was collected from the State and District Health authorities and entered into Excel and extracted for coding and analysis. The structured observations data was entered into EpiInfo (8) and extracted into Excel for coding, cleaning, and analysis. Ethics approval for the research was obtained from the Institutional Ethics Committee of the Public Health Foundation of India (Reference # TRC-IEC-342.1/17) along with approvals from the Indian Health Ministry’s Screening Committee (2017–2,250).

## Results

**Figure 1:**
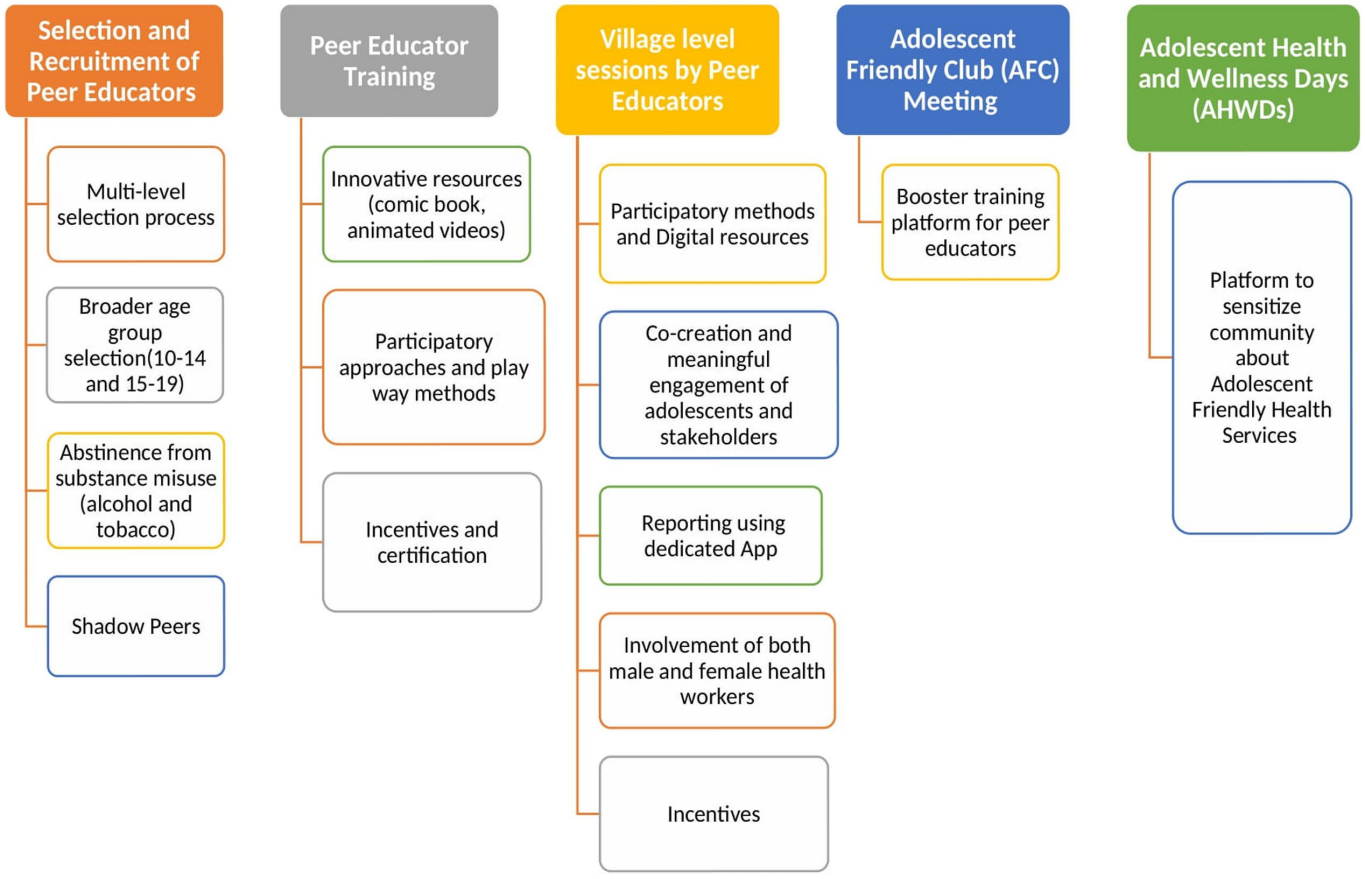
Recommendations for scalingup the peer education programme.

The Peer Education programme, AFC and AHWDs exhibited both similarities and variations in implementation across the study states. These variations were influenced by *contextual factors* (deviations from the Operational Framework of National Adolescent Health Strategy prompted by COVID-19) and the *model of implementation*. These variations contributed to the identification of barriers and facilitators across programme activities as listed in Table 2. This section also highlighted the strategies for strengthening Peer Education Programme implementation as reported by the stakeholders (Figure 1).

**Table 2:** Key learnings from the implementation of peer education programme under RKSK.

Programme activities	Madhya Pradesh	Maharashtra	Facilitators	Barriers
Selection and recruitment of peer educators				
Number of peer educators	Two PEs per 1,000 population/ASHA habitation	Four PEs per 1,000 population/ASHA habitation	Representative coverage of adolescent groups with a broader age bracket of PEs.	Skewed distribution if older PEs selected
Age of peer educators	15 years and above	10–14 years and 15–19 years		
Criteria for selection	Life skills like leadership, communication, friendly, knowledgeable, and responsible. Abstaining from substances like tobacco, or alcohol	Life skills like leadership, communication, friendly, knowledgeable, and responsible.	Additional selection criteria along with life skills ensure unbiased selection. PEs are seen as role models among their friends	
Mechanism for coping with attrition	Two shadow peers (male and female) from 10–14 years		Provides programme continuity	Recruitment and training dependency on funding availability
Peer educator training				
Training Process (days, timings)	Structured days, duration and format	Flexible days, timings and format	The unstructured format enables PEs to learn about contemporary issues beyond the RKSK themes	An unstructured format results in missed opportunities to discuss a few topics.
Master Trainers	Dedicated HR Human Resources (NGO Trainer Mentor)	Master trainer (Medical Officer, ANM) and any other health worker (ASHA facilitator, Community Health Officer)	Standardised information delivered by dedicated staff	Lack of continuous supportive supervision due to the involvement of other staff
Resources and Approach	Standardised and prescribed resources like RKSK Manual, PE training Kit, posters, innovative IEC-like comic books, and videos Use of participatory methods like role plays and case studies	Limited use of RKSK resources due to its unavailability	The utilisation of prescribed resources and participatory methods fosters increased interaction and engagement among adolescents	Lack of prescribed resources
		Limited use of participatory methods		Limited engaging strategies
Training Assessment	Pre -post evaluation	No Pre-Post evaluation undertaken	Suggest the key areas for strengthening	
Leveraging resources from other health programmes	School Health Programme manual under AYUSHMAN BHARAT	Adolescent Reproductive Sexual Health programme manuals	Convergence of resources enhanced learning of peer educators beyond RKSK themes	
Incentives	Travel allowance, Certificate of Completion of training	Travel allowance	Motivates PEs	
Village level sessions by peer educators				
Supportive Supervision	Dedicated male and female HR (NGO Trainer Mentor)	ASHA and sometimes PEs conduct sessions alone	Maintaining the regularity of sessions	
Reporting Mechanism	Mobile App	No reporting mechanism	Ensures maintaining session regularity	
Resources and Approach	Use of comic books, videos, and play-way methods like role plays and case studies	Transaction of information during the session relied on knowledge, notes, and Google for information due to limited availability of hard copies of RKSK resources	Participatory methods and innovative resources result in more interaction and engagement during the session	Use of less engaging strategies and resources
Incentives	Non-financial incentives, Additional scores through Continuous and Comprehensive Evaluation (CCE)	Non-financial incentives	Additional scores through CCE Strategic convergence between the education and health departments	Parental hesitancy
Adolescent Friendly Club (AFC) meetings				
Frequency	Monthly at sub centre	Monthly at sub-centre	Platform for the handholding of PEs	
Resources and strategies	Comic books, Peer Job Aids, Role plays	No specific RKSK resources but the use of other programme resources (National Mental Health programme)	Participatory methods and innovative resources result in more interaction and engagement	Less interaction and engagement due to less engaging strategies and resources to facilitate these meetings
Adolescent Health and Wellness Days (AHWDs)				
AHWDs	On hold due to COVID-19	Ongoing	A unique platform sensitising the community about adolescent health issues and the availability of services	Limited parental and community participation

### Peer education programme

#### Recruitment and selection of peer educators

As per the National Adolescent Health Strategy (2014), each village with a population of 1,000 per ASHA habitation is required to select four PEs (2 boys and 2 girls), aged between 15 and 19 years. The selection is typically conducted through a community-level event or the Village Health, Sanitation and Nutrition Committee. ASHA coordinates the selection with other decision-makers grounding their decision on personality traits of adolescents like strong motivation, entrepreneurism, leadership qualities etc. (1).

The study findings spotlighted that following a *multi-level selection process*, two PEs from Madhya Pradesh and four PEs from Maharashtra were selected. In Madhya Pradesh, the selection process involved the NGO Trainer Mentor in consultation with ASHA and a Village Health, Sanitation and Nutrition Committee. Conversely in Maharashtra, ASHA took the lead in selecting PEs with support from ANM, ASHA Facilitator, Community Health Officer, Medical Officer and/or the school teacher. The inclusion of teachers in the selection process demonstrated the intersectoral convergence between health and education in the programme activities.

*“All adolescents were gathered by ASHA, confirming their age (15–17 years) and skills, qualities like leadership, someone who is cooperative, talks nicely, not arrogant, not short-tempered, and does not use tobacco, or alcohol being selected as PE. We have been told by the district that we should not select the ASHA or ASHA facilitator’s child as PEs.” NGO Trainer Mentor, Panna, Madhya Pradesh.*

*“With the help of ASHA, we went to different schools to inform adolescents about this programme. Among these students, the ones who were active, participative, took responsibility and were able to talk to others, were selected. It was also important for them to be friendly and knowledgeable.” ANM, Nashik, Maharashtra.*

Variability was evident in the eligibility criteria for PEs’ selection. Maharashtra allowed *both younger (10–14 years) and older adolescents (15–19 years)* to possess skills like leadership, communication, friendliness, knowledge, and responsibility. In Madhya Pradesh, adolescents aged 15 years and above were considered, with an additional criteria of abstaining from substances like tobacco, or alcohol and no familial relationships with community health workers.

To address the issue of PEs attrition, Madhya Pradesh employed a unique strategy involving *Shadow Peers*, consisting of one boy and one girl, aged 10–14 years from the adolescent group. These Shadow Peers support trained PEs by accompanying them in all peer-led activities. Additionally, if a trained PE discontinues the programme, the Shadow Peers step in to ensure programme continuity.

*“We select shadow peers from younger age groups (10–14 years) to avoid discontinuity in the programme, in case if Peer Educator leaves the programme.” NGO Representative, Madhya Pradesh.*

Attrition of peer educators (PEs) in both states was predominately associated with factors such as adolescent girls getting married, relocation for higher education, and parents perceiving the programme as not beneficial or a waste of time for their children. This perception could have arisen due to the lack of understanding among parents about their child’s roles and responsibilities in the programme, leading to hesitancy to send children for programme activities, along with inadequate incentives.

*“Parents hesitate and they ask what will be the benefit of this programme. They think that their child’s studies will be affected after enrolling in the programme.” NGO Trainer Mentor, Damoh, Madhya Pradesh.*

*“Sometimes parents do not allow their children to become peer educators. If they are school students, they miss their school and if they are working, they are not able to work.” ASHA, Nashik, Maharashtra.*

#### Training of peer educators

PEs are mandated to undergo 6 days of training following the structured session plan outlined in the Peer Educators’ Training Manual. The training is conducted by Medical officers, ANMs or NGO Trainer Mentors who receive training at the Regional level. A standard training batch consists of 40 participants, including 32 PEs and 8 ASHAs.

Drawing from observed training batches (*n* = 4), Madhya Pradesh (*n* = 2, 6 days each); and Maharashtra (*n* = 2, 4–5 days each), several distinctions were revealed in the training regarding duration, resources, master trainers, incentives and training assessments. In Madhya Pradesh, the training was structured with fixed days (*n* = 6 days), held over consecutive weekends (six Sundays) or Gazetted holidays from 10 am- 4:30 pm. Whereas, in Maharashtra, trainings were more flexible and ranged from four to five consecutive weekdays. Due to the flexible training structure, certain topics like violence, leadership skills, nightfall etc. were missed. However, the flexibility in the training schedule allowed the inclusion of many contemporary issues in addition to the six priority themes of RKSK, such as social media usage, personal and community hygiene, cybercrime and the importance of planting trees.

*There was an introduction about adolescence and then topics related to the six themes of RKSK were covered over 6 days, topics like nutrition, sexual and reproductive health, non-communicable diseases, GBV, substance use and mental health. ASHA, Damoh, Madhya Pradesh.*

In Madhya Pradesh, the training sessions were led by a dedicated human resource, NGO Trainer Mentor. These sessions utilised standardised and prescribed resources including PE Training Manual, PE Activity Book, game cards (Kranti Bhranti cards), innovative IEC-like comic books, and animated videos covering RKSK themes and subthemes (9).

Regarding the deployment of the strategies, Madhya Pradesh used participatory methods like role plays and case studies. Additionally, the provision of a PE Training Kit, containing a PE Activity Book, bag, PE records formats, pen, caps, jacket, and umbrella was observed as per the operational framework.

In both implementation models, effective integration and utilisation of resources from other national health programmes were observed. Maharashtra utilised the Adolescent Reproductive Sexual Health manual, while Madhya Pradesh, leveraged the School Health and Wellness Programme manuals under Ayushman Bharat.

As per the routine RKSK data collected, the pre-post training assessment (*n* = 20) of PEs was conducted only in Madhya Pradesh. In Panna, knowledge scores increased from 9.6 to 19.9, while in Damoh, the score increased from 7.7 to 14.5 (out of 20 total scores).

Regarding incentives, in addition to Travel and Dearness Allowance, a training completion certificate was provided to the PEs as an incentive only in Madhya Pradesh if PE completes a minimum of 6 days of training.

In Maharashtra, PE training was conducted by Master Trainers (ANM or Medical Officer) or other health workers based on their availability, often leading to limited supportive supervision due to their involvement in other programmes.

Regarding the use of strategies, the traditional educational approach used in Maharashtra relied mainly on lectures, and lacked interactive and participatory activities, without the provision of a PE kit. This approach limited adolescent engagement as observed.

The reported barriers to attending training sessions by PEs reported by the participants were inadequate access to public transportation, inappropriate weather conditions, remote training locations, and conflict of training schedule with school activities.

*There was a transportation problem as they (PEs) had to come from different and faraway places and there was no proper transportation available. ASHA Facilitator, Nashik, Maharashtra.*

*Many Peer Educators could not attend the training as it (the training venue) was in the hilly region and PEs were unable to attend due to lack of transport. NGO Trainer Mentor, Panna, Madhya Pradesh.*

*The weather was a major challenge. During the rains, we had to suspend the training. NGO Representative, Damoh, Madhya Pradesh.*

*Teachers were not letting them (peer educators) miss their school as they had exams so they said they could not come for the training. ASHA, Nashik, Maharashtra.*

*When we talk about sexual organs, intercourse, they feel embarrassed. When we talk about the usage of condoms, girls feel embarrassed. ASHA, Nashik, Maharashtra.*

#### Village-level PE sessions

As per the operational framework, selected and trained male and female PEs lead weekly, participatory sessions lasting 1 to 2 h with their adolescent group using PE kits. These include a PE Activity book (eight modules and 15 sessions) and game cards (a fun way to learn about adolescent health). The frequency and duration of PE sessions differed in both models. In Maharashtra, the reported frequency of sessions ranged from monthly to quarterly, with durations ranging from 45 to 120 min. Whereas in Madhya Pradesh, the sessions were held monthly, lasting from 30 to 90 min.

*“Once every 2 months, I take a session and share health information.” Male PE, Nashik, Maharashtra.*

The attendance at these sessions ranged from 24 to 49 adolescents in Maharashtra and 19–48 adolescents in Madhya Pradesh, as observed by the study team. In Madhya Pradesh, the regularity of sessions was maintained due to dedicated male–female NGO Trainer Mentors for supportive supervision. In Maharashtra, there is a lack of dedicated supportive supervision. ASHAs supervised sessions, and at times, PEs conducted sessions independently due to ASHA engagement in other health programmes. This has led to irregular sessions, despite programme guidelines recommending weekly sessions.

*“It (PE Session) happens once a month for 2 h. I and ANM are present at the meeting. But if ANM does not want to conduct the meeting, I conduct it. Peer educators and adolescent boys and girls are present at that time.” ASHA, Yavatmal, Maharashtra.*

*“We go to monthly sessions and support them. We help the PE if they are stuck somewhere or cannot answer a question.” NGO Trainer Mentor, Damoh, Madhya Pradesh.*

*“If an adolescent is facing any mental health issues and if we can make him overcome through the programme, that I think is the most challenging part.” ASHA Facilitator, Nashik, Maharashtra.*

During sessions in Madhya Pradesh, PEs utilised a variety of RKSK resources, including the PE Activity Book*, Kranti Bhranti* cards (game cards), comic books, and interactive strategies like role plays, videos and case studies to engage with the adolescents. Most PEs reported enjoying comic books, games and attending sessions on common topics like nutrition and non-communicable diseases. Due to the limited availability of hard copies of RKSK resources in Maharashtra, the transaction of information by PEs to their group primarily relied on their knowledge, personalised information, self-developed notes, and the internet. An innovation was introduced to maintain records of village-level sessions in Madhya Pradesh through an RKSK MP App., accessible via mobile. This application was developed to allow NGO mentors to upload details of their supportive supervision, including the session’s name, village name and time spent by them in the village (entering and exit time) etc.

The reported barriers related to adolescents’ footfall at these sessions ranged from conflict with schools’ activities, lack of incentives, less engaging strategies, parents’ unwillingness, gender of the health workers (mostly female) and difficulties encountered by PEs in implementing these sessions. It was reported that discussing sexual and reproductive health issues, such as sexual organs, intercourse, and usage of condoms made adolescents uncomfortable, especially in mixed-gender settings whereas, topics like nutrition, and non-communicable diseases, were comfortably discussed. Additionally, as reported by ASHA Co-ordinators, discussing mental health issues also posed a significant challenge among PEs, and there was a notable consensus on the necessity for specialists or experts to provide training to PEs, on adolescent mental health issues.

*It is challenging to gather adolescents on days when schools and colleges are functional. Peer Educator, Panna, Madhya Pradesh.*

*Some adolescents do not come at all; there should be something to attract them, like offering them refreshments to attend sessions. NGO Trainer Mentor, Damoh, Madhya Pradesh.*

*When they (adolescents) are told something that they should do, they are not interested. When PE uses songs, sports/ games in the sessions, adolescents become excited. ASHA, Yavatmal, Maharashtra.*

*Boys engage in PE sessions less since the ANM, ASHA, and Block facilitators are all females. District Official, Yavatmal, Maharashtra.*

*When we talk about sexual organs and intercourse, they (adolescents) feel embarrassed. When we talk about the usage of condoms, girls feel embarrassed. ASHA, Nashik, Maharashtra.*

*Sexual and reproductive health. They feel shy and do not want to talk about it. PE, Nashik, Maharashtra.*

In Maharashtra, sessions on personal hygiene were particularly favoured among adolescents, potentially influenced by the COVID-19 pandemic context. Sessions on menstruation were most favoured among adolescent girls. Across all groups, the least liked session was on child marriage. Comparatively, in Madhya Pradesh, personal hygiene remained the top choice, while sessions on menstruation, were preferred by female adolescents, and undernutrition and anaemia among boys. The least favoured sessions were pubertal changes.

### Adolescent friendly club

In Madhya Pradesh, AFC are established at the sub-health centre level to provide handholding to PEs, guided by ANMs, covering five villages or a population of 5,000 and 10 to 20 PEs per club. AFCs are expected to convene monthly to discuss PE sessions, address PEs’ concerns or questions, plan upcoming sessions, AHWD, and activities (drawing competitions, skits, quizzes and debates). Stakeholders’ discussions revealed that AFCs were put on hold from March 2020 but resumed in May 2022 in Madhya Pradesh. Whereas in Maharashtra, AFC continued operating at the sub-centre level.

The commonality between both states was the consistent support provided to PEs, utilising AFCs as a platform for training untrained PEs. As observed (n = 8, Madhya Pradesh: 4, Maharashtra: 4), attendance at the AFCs ranged from 8 to 18 PEs (Male: 4–8; Female: 4–10) in Madhya Pradesh and 7–17 PEs (Male: 1–7; Female: 6–11) in Maharashtra.

*Peer Educators discuss their problems and we together plan for the next month. ANM, Yavatmal, Maharashtra.*

A diverse set of workforces was engaged in organising AFC meetings in both states. In Maharashtra, ANMs, Community Health Officers, ASHAs, ASHA, Block Facilitators, RKSK Counsellors, Multi-Purpose Workers (if available) were employed, while in Madhya Pradesh, ANM and NGO Trainer Mentor were involved. Observations revealed that the workforce in Madhya Pradesh utilised strategies and resources like RKSK manuals, comic books, case studies and role plays. However, in Maharashtra, ANMs and Community Health Officers utilised resources from other national programmes (e.g., Mental Health Programme).

The participants from Maharashtra reported several barriers specific to AFCs, including a view that the strategies were not sufficiently interactive and engaging, along with a lack of resources to support these meetings.

### Adolescent health and wellness days

According to operational guidelines, AHWDs, formerly known as Adolescent Health Days are scheduled to be held quarterly in every village at Anganwadi Centre or community space. The aim is to raise awareness among adolescents, parents, families and stakeholders about the determinants of adolescent health, and improve awareness of adolescent health-related services, particularly Adolescent Friendly Health Clinics/helplines. During AHWDs, PEs, ASHAs, AWWs, and other stakeholders (including NGOs where present) are supposed to mobilise adolescents, parents, and other community members to gather at the nearest Anganwadi Centre or community space and organise various “infotainment” activities. RKSK stakeholders reported that the implementation of these days in Madhya Pradesh was suspended in March 2020 due to the COVID-19 pandemic and budgetary constraints.

*AHWDs create awareness and build confidence in youngsters to visit the clinic and have trust in the counsellor. There were no days organised during COVID-19, so they have less knowledge about the Adolescent Friendly Health Clinics. Counsellor, Adolescent Friendly Health Clinics.*

*Previously (before COVID-19), we used to organise AHWDs like a health mela in every village, by involving everybody. People felt there is a clinic to support them. Since the AHWDs stopped, a lot of people are not aware of the clinic and they think the clinic is also closed. NGO Trainer Mentor, Panna, Madhya Pradesh.*

In contrast, AHWDs resumed in Maharashtra after a gap of 5–6 months post-first lockdown but on a reduced scale. Observations revealed that in Maharashtra (*n* = 4), the attendance of adolescents during these AHWDs ranged from 24–70. Notably, none of the observed AHWDs in Maharashtra were conducted following the RKSK operational framework. Families, parents, village gatekeepers (such as Sarpanch), or Village Health, Sanitation and Nutrition Committee lacked information about these days. The commodity and clinical services were also lacking in all executed AHWDs including Body Mass Index screening (*n* = 3), anaemia testing (*n* = 1), diabetes testing (*n* = 4), provision of IFA tablets (*n* = 2), referrals to Adolescent Friendly Health Clinics (*n* = 4).

The low awareness about RKSK and Adolescent Friendly Health Clinics in Madhya Pradesh is attributed to the halting of AHWD. This has resulted in a decline in the number of referrals to clinics, as reported by the counsellor of Adolescent Friendly Health Clinics.

*No, I am not aware about any programmes or Adolescent Health Days. I have not gone to anything other than an Anganwadi on Tuesdays. Adolescent Boy, Yavatmal, Maharashtra.*

## Discussion

The “i-Saathiya” study findings offer valuable insights into the implementation of the Peer Education programme within the RKSK, highlighting the uniqueness of two existing models in India. Our findings provide insights into barriers and facilitators influencing the peer education programme, relevant for designing peer interventions within national adolescent health programmes, globally. They complement prior evidence on the need to strengthen peer education programme (10). These insights are crucial for enriching national and global literature and prioritising adolescent health in SEAR countries, as well as globally.

### Facilitators influencing peer education programme

#### Peer educator selection criteria and multi-level recruitment process

The ‘i-Saathiya’ findings highlighted that having four PEs in Maharashtra resulted in wider and consistent coverage, encompassing both younger and older adolescents. In a similar vein, a successful study in Ethiopia, which involved 25 PEs per 5,000 population (4 PEs per 1,000 population) within its Youth Friendly Services, showed that 78% of participants were aware of PEs and their activities (11).

Selecting appropriate PEs is crucial, as they play a pivotal role in disseminating information and fostering positive behaviours among peers. Our findings showed that in both states, PE selection was based on multiple-set criteria to maintain integrity of the selection process. This contrasts with another Indian study, where PEs were randomly selected at PHC (12). Literature reveals that several peer-led programmes choose PEs who volunteered for their roles (11, 13). However, studies have shown positive results, particularly in terms of percentage increase in knowledge, while employing a multi-criteria selection process for choosing PEs, as in our study (14, 15). Notably Madhya Pradesh’s additional criterion of refraining from substance misuse for PE selection holds a significant potential for wider implementation across India and the region. This inclusion criterion aligns with the operation manual of “Targeted Intervention (TI) for Substance Use Prevention in community—An Operational Manual on Community-Based Peer-Led Intervention” (CPLI) (16). The selection of PEs with no familial relationships with community health workers as in Madhya Pradesh ensures equal opportunities for all adolescents to be selected as PEs although this is not formally evaluated.

#### Incentivisation of peer educators

Both states offered non-financial incentives to acknowledge the contributions of PEs. In Madhya Pradesh, noteworthy efforts of additional recognition and scores through the education department were granted, showcasing strategic convergence between the education and health departments to incentivise PEs. Similar linkages could be explored with the Ministry of Skill Development and Entrepreneurship. Similar to India, in other countries, like Zanzibar, the Zanzibar Family Planning Programme provides bicycles and other equipment to PEs supporting their work as community-based distributors. PEs are permitted to rent out the equipment when not in use, supplementing their income (17). In Kenya, the Y-PEER initiative has established annual awards to promote and honour their outstanding contributions (17). Supporting the appreciation of incentives, a study in Zimbabwe noted that PEs dedication to project tasks declined following the discontinuation of financial rewards (18).

#### Use of participatory approaches and booster trainings

Study participants reported that the use of participatory methods like role plays and case studies were effective in engaging them. This approach has been successful in various peer-led interventions in countries like Turkey (19) and United States (20).

The utilisation of participatory methods, alongside RKSK resources and digital tools rendered the Adolescent Friendly Club meeting more captivating. Since PEs under the programme receive training only once, these meetings serve as a valuable opportunity for booster training sessions, helping to refresh their knowledge and skills. Booster training has been identified as essential (4, 21) and studies also highlighted their significance (21).

#### Inter-ministerial and inter-sectoral collaborations

In both states, resources from other national health programmes were leveraged, which led to broader coverage of topics and highlighted the effectiveness of inter-ministerial linkages in augmenting the programme’s impact on its beneficiaries. In Maharashtra, reliance on ANMs and Medical Officers for training, with other health workers filling in, led to non-standardised information dissemination and accountability issues. Evidence from Vietnam (22), Indonesia (23), Kenya, Madagascar and South Africa (24) displays the use of external organisations in a social franchise model for improving the quality of healthcare services for adolescents. However, countries like the United Kingdom (25) and New South Wales, Australia (26) have even adopted federal models for providing youth-friendly services.

#### Frequent peer educator sessions

In both states, sessions by PEs occurred monthly to quarterly, but the i-Saathiya study findings suggest increasing the frequency to fortnightly to foster behaviour change. Studies have demonstrated that improved behaviour change is often achieved through weekly (27) or fortnightly (13) sessions.

#### Reviving adolescent health and wellness days

Both beneficiaries and their parents exhibited unfamiliarity with Adolescent Health and Wellness Days. Reviving and organising these days can raise awareness among adolescents (28, 29) and the community, promoting existing health services such as Adolescent Friendly Health Clinics and helplines. This strategy would help create a supportive environment for PEs contributing to the programme’s sustainability.

### Barriers influencing the peer education programmes

#### Attrition among peer educator’s

Peer-led programmes often face significant turnover challenges due to migration, marriage of female PEs etc. (30), as reported by study participants. To address attrition, an innovative approach was the inclusion of ‘Shadow Peers Educators’ in Madhya Pradesh. While Shadow PEs lack formal training, they acquire learning through observations and experiential learning while accompanying trained PEs. This approach mirrors the method commonly used in clinical settings, where the learner gains valuable insights through exposure to real-life situations (31).

#### Hesitancy in discussing sensitive topics

There was hesitancy when discussing sensitive topics like sexual and reproductive health issues, especially in the presence of health workers. To address this, proposed solutions include co-creating digital resources with adolescents and service providers, including short reels, WhatsApp messages, and videos which are promising (32) and cost-effective (33). These resources have proven to be effective in promoting health and encouraging behaviour change (34). Digital interventions are beneficial as they are easily accessible, engaging (35) and overcome barriers like gender and literacy (36). Emerging evidence from public health research indicates that co-creating interventions are more likely to create feelings of ownership among stakeholders, increase stakeholder buy-in for implementation, lead to better-tailored solutions (37), enhance the acceptability and feasibility of the intervention, and maximise its likelihood of longer-term sustainability (38). Before a country adopts a digital approach, it is important to address the associated issues like cyberbullying, sexual grooming and exploitation, trafficking, and child pornography, within the national adolescent health programme (30). We may also need to consider the digital access disparities existing in India.

While the Peer education approach effectively enhances knowledge and behaviours among adolescents (39, 40). However, its success hinges on several factors, including, settings, context, and participants’ values. Therefore, designing and implementing a Peer Education Programme necessitate well-defined strategy documents outlining key activities, training, supervision, and robust monitoring and evaluation mechanisms. Across all these activities, it is important to promote adolescent participation and decision-making through meaningful engagement. Adolescents can provide invaluable insights into the unique challenges they face in their specific context and can act as change agents by championing the strategy among their peers. The study has few limitations, the cross-sectional design nature of the current study limits establishing definitive cause-and-effect relationships. Further, to effectively evaluate the programme’s long-term impact on adolescent health behaviours, future research should utilise longitudinal designs, enabling statistically stronger and more reliable conclusions. It is also important to acknowledge that part of this study was conducted during the COVID-19 pandemic, which influenced the implementation of certain activities compared to routine programme implementation.

## Conclusion

The findings of the i-Saathiya study necessitate the need to address identified barriers to strengthen the implementation of the peer education programme and enhance its acceptance among adolescents. The insights gleaned offer valuable guidance for policymakers, practitioners, and stakeholders involved in RKSK’s planning and implementation. These identified gaps can offer valuable lessons to the study states as well as for other states, aiding in strengthening programme guidelines. These findings not only provide a pathway to strengthen the Peer Education programme but also offer guidance to other countries employing the peer education model.

## Data Availability

The raw data supporting the conclusions of this article will be made available by the authors, without undue reservation.
