# Supplementary material for: Implementation outcomes of peer education programme comparing state-led and NGO-facilitated models in two Indian states: qualitative findings
Source: Front Public Health. 2024 Nov 1;12:1434959. doi: 10.3389/fpubh.2024.1434959 (PMC11564149; doi:10.3389/fpubh.2024.1434959)
Supplement: Supplementary file 1 [file Table_1.docx]

**Supplementary Table 1: An overview of the themes covered during IDIs and FGDs**

| **S.No** | **Themes and sub-themes** | **In-Depth Interviews (IDIs)** | **Focus Group Discussions (FGDs)** |
| --- | --- | --- | --- |
| **1** | **Implementation of the RKSK’s Peer Education program** | | |
| 1.1 | Recruitment and Selection of Peer Educators | √ |  |
|  | Number of Peer Educators selected per population | √ |  |
|  | Selection and Eligibility Criteria of Peer Educators | √ |  |
|  | Who is responsible for selection? | √ |  |
|  | Challenges and Facilitators in Peer Educator Selection | √ |  |
|  | Any State-level innovations | √ |  |
|  | Incentives for Peer Educators | √ |  |
|  | Recommendations for strengthening the recruitment and selection process | √ |  |
| **1.2** | **Peer Educator Trainings** | | |
|  | Structure of Training | √ |  |
|  | Training schedule and Venue | √ |  |
|  | Training Frequency and Duration | √ |  |
|  | Training curriculum, resources and topics covered | √ |  |
|  | Master trainer/supportive supervision | √ |  |
|  | Mode of Training | √ |  |
|  | Strategies used during training | √ |  |
|  | Training assessment | √ |  |
|  | Incentives to attend the training (*types of incentive*, *frequency of incentives provides)* | √ |  |
|  | Barriers in attending training sessions | √ |  |
|  | Barriers to conducting PE training sessions | √ |  |
|  | Facilitator | √ |  |
|  | Recommendations for strengthening | √ |  |
| **1.3** | **Village-level Peer Educator sessions** | | |
|  | Frequency and Duration of sessions | √ | √ |
|  | Average attendance during these sessions | √ |  |
|  | Supportive Supervision | √ |  |
|  | Themes covered during the sessions | √ | √ |
|  | Most liked topic by the adolescents | √ | √ |
|  | Most disliked topic by adolescents | √ | √ |
|  | Any innovation | √ |  |
|  | Trainings Resources used by peer educators during the sessions | √ |  |
|  | Barriers/Challenges in conducting the sessions/during the session | √ |  |
|  | Barriers/Challenges attending the sessions |  | √ |
|  | Recommendations for strengthening | √ | √ |
| **1.4** | **Adolescent Friendly Club (AFC)** | | |
|  | Purpose of conducting | √ | √ |
|  | AFC schedule and Frequency | √ | √ |
|  | Workforce involved in conducting AFC | √ | √ |
|  | Activities conducted during AFC | √ | √ |
|  | Barriers to conducting AFC | √ |  |
|  | Facilitators in conducting AFC | √ |  |
|  | Any state-level innovation | √ |  |
|  | Recommendations for strengthening | √ | √ |
| **1.5** | **Adolescent Health and Wellness Days (AHWDs)** | | |
|  | Schedule and frequency | √ | √ |
|  | Activities conducted at AHWDs | √ | √ |
|  | Facilitators to implementation of AHWDs | √ |  |
|  | Barriers to implementation of AHWDs | √ |  |
|  | Any state-level innovation | √ |  |
|  | Recommendations for strengthening | √ | √ |
